# Modification of Growth, Yield, and the Nutraceutical and Antioxidative Potential of Soybean Through the Use of Synthetic Biostimulants

**DOI:** 10.3389/fpls.2018.01401

**Published:** 2018-11-08

**Authors:** Agnieszka Szparaga, Sławomir Kocira, Anna Kocira, Ewa Czerwińska, Michał Świeca, Edmund Lorencowicz, Rafał Kornas, Milan Koszel, Tomasz Oniszczuk

**Affiliations:** ^1^Department of Agrobiotechnology, Faculty of Mechanical Engineering, Koszalin University of Technology, Koszalin, Poland; ^2^Department of Machinery Exploitation and Management of Production Processes, Faculty of Production Engineering, University of Life Sciences in Lublin, Lublin, Poland; ^3^Institute of Agricultural Sciences, State School of Higher Education in Chelm, Chelm, Poland; ^4^Department of Biomedical Engineering, Faculty of Technology and Education, Koszalin University of Technology, Koszalin, Poland; ^5^Department of Biochemistry and Food Chemistry, Faculty of Food Science and Biotechnology, University of Life Sciences in Lublin, Lublin, Poland; ^6^Department of Thermal Technology and Food Process Engineering, Faculty of Production Engineering, University of Life Sciences in Lublin, Lublin, Poland

**Keywords:** antioxidant activity, foliar application, *Glycine max*, growth, nutrients, titanium, phenols, yield

## Abstract

Improvement of crop cultivation technologies is focused on increasing crop productivity and improving yield quality, and at the same time on minimizing risks posed to the natural environment. The use of biostimulants contributes to the increase in the productivity of plants, especially under their exposure to stress induced by negative environmental stimuli. A field experiment was conducted in three growing seasons (2014–2016). Seeds of soybean of the Atlanta cultivar were sown in the third decade of April. Two synthetic biostimulants were used in the growing period in the form of single (stage BBCH 13-15) or double (stage BBCH 13-15, BBCH 61) spraying: Atonik (in concentrations of 0.1 and 0.2%) and Tytanit (in concentrations of 0.07 and 0.13%). Atonik, the first tested biostimulant, contained three phenolic compounds: sodium p-nitrophenolate, sodium o-nitrophenolate, and sodium 5-nitroguaiacolate. The Tytanit preparation contained a titanium complex, magnesium oxide, and sulfur (VI) oxide. This work presents a complex study addressing the action of the biostimulants Atonik and Tytanit and demonstrates their effect on the physiological traits, plant productivity, and seed yield quality of *Glycine max* L. The conducted experiment proved that the biostimulant type, as well as the number of its applications and its concentration, modified the biometric traits, crop productivity, as well as yield quality and the nutraceutical and antioxidative potential of soybean seeds. It was also found that by positively affecting plant growth and seed yield, the Atonik and Tytanit also resulted biostimulants decreased the protein and lipid contents in seeds. A double application of these preparations in their higher concentrations had a more positive impact on soybean seed number and soybean seed yield. The use of both Atonik and Tytanit resulted also in an increased antioxidative activity of soybean seeds. The greatest increase in this activity was observed after the application of the Tytanit preparation. Dietary fiber fraction analysis demonstrated an increase in the acid-detergent fiber, lignin, and cellulose contents in soybean seeds as a result of biostimulant application; however, the increase was greater upon the use of Tytanit. In contrast, the neutral-detergent fiber, cellulose, and hemicellulose contents were observed to decrease in all analyzed combinations of crops treated with the tested preparations.

## Introduction

Modern agriculture tends to minimize the use of mineral fertilizers and chemical plant protection agents that are replaced by preparations of natural origin (Maciejewski et al., 2007). This group of preparations includes biostimulants whose one of the basic tasks is to alleviate environmental stress (Yakhin et al., 2017).

The phrase “biostimulant” is increasingly used in scientific literature (Du Jardin, 2012; Calvo et al., 2014; Halpern et al., 2015). The first definition of biostimulants was proposed by Kauffman et al. (2007), who described them as plant growth promoters. This definition has been evolving ever since, and according to Du Jardin (2012), “*plant biostimulant is any substance or microorganism, in the form in which it is supplied to the user, applied to plants, seeds or the root environment with the intention to stimulate natural processes of plants to benefit their nutrient use efficiency and/or their tolerance to abiotic stress, regardless of its nutrients content, or any combination of such substances and/or microorganisms intended for this use.”*

Despite the establishment of the European Biostimulants Industry Council (EBIC) (formed to develop the legal regulations concerning the registration of biostimulants according to the specificity of their action), the registration of preparations is still based on the legal regulations set for fertilizers and plant protection agents (Traon et al., 2014; Chojnacka, 2015; Du Jardin, 2015).

Treatment of plants with preparations containing active compounds may foster many unquestionable advantages. Not only do such preparations support the growth and development of plants, but their application leads to cost reduction and increased effectiveness of crop fertilization (Brown and Saa, 2015; Van Oosten et al., 2017). Their multiple advantages also include reduced incidence of some noninfectious diseases induced by nutrient deficiency (Liakas et al., 2006; Jakiene, 2013). The effectiveness of biostimulants is determined by many factors, including the appropriate choice of preparations, their dose, concentration, and methods of application, as well as the species and cultivar of plants and environmental factors (Grabowska et al., 2012; Kolomaznik et al., 2012).

Ensuring effective protection of crops against biotic and abiotic factors in agricultural practice is difficult; hence, the use of synthetic or natural biostimulants is recommended. Their task is to improve biochemical, morphological, and physiological processes in a crop under its exposure to stresses induced by negative stimuli (Basak, 2008; Paradiković et al., 2011; Du Jardin, 2012; Calvo et al., 2014; Posmyk and Szafranska, 2016). The group of natural biostimulants includes preparations based on free amino acids, extracts from marine algae and fruits, effective microorganisms, and also humic compounds and chitosan (Calvo et al., 2014). In turn, synthetic preparations contain mainly plant growth regulators, polyphenolic compounds, and such plant stimulants as inorganic salts or essential elements (Du Jardin, 2012; Przybysz et al., 2014).

Among the synthetic biostimulants, special attention is given to the preparations with commercial names Atonik and Tytanit. The active compounds of the first preparation, also referred to as Asahi SL or Chapperone, include three phenolic compounds (Table 1), which are now registered in the European Union as pesticides (URL: http://ec.europa.eu/food/plant/pesticides/eu-pesticides-database/public/). Although Atonik has been used for many years in the cultivation of major crops worldwide, opinions on its effects vary. Depending on the plants tested, analyses demonstrated either positive or negative effects of its application on yield quality (Kozak et al., 2008; Malarz et al., 2008; Przybysz et al., 2014; Kocira et al., 2015b,c, 2017a; Szczepanek et al., 2017a,b). The second mentioned preparation, Tytanit, generally contains a titanium complex (Table 1). At present, Tytanit is registered in the European Union as a fertilizer (https://trademarks.justia.com/791/78/tytanit-79178603.html). Its formula was developed and implemented into agricultural practice in Central and Eastern Europe with the aim to improve plant productivity by stimulating activities of selected enzymes, increasing chlorophyll content, stimulating photosynthesis, promoting uptake of nutrients, increasing tolerance to stress, and improving yield quality (Lyu et al., 2017). Titanium, being its constituent, is perceived as an element beneficial for plant growth and development; however, mechanisms underlying these positive effects still remain unclear (Ghooshchi, 2017; Lyu et al., 2017).

**Table 1:** Plant developmental stages and dates of application of biostimulants.

**Biostimulant**	**Formulation**	**Number of sprays and plant developmental stages in which the biostimulants were applied**	**Concentration**	**Volume of working solution/working pressure**	**Date of spraying**		
					**2014**	**2015**	**2016**
Atonik	sodium p-nitrophenolate NaC_6_H_4_NO_3_ (3.75 g/L), sodium o-nitrophenolate NaC_6_H_4_NO_3_ (2.5 g/L), sodium 5-nitroguaiacolate NaC_7_H_6_NO_4_(1.25 g/L); dissolved in water	Single spraying BBCH 13-15 (LSS)	0.1%	300 l·ha^−1^/0.30 MPa	June 21	June 20	June 7
		Single spraying BBCH 13-15 (HSS)	0.2%				
		Double spraying BBCH 13-15, BBCH 61 (LDS)	0.1%		June 21, July 5	June 20, July 3	June 7, June 23
		Double spraying BBCH 13-15, BBCH 61 (HDS)	0.2%				
Tytanit	Ti as titanium ascorbate (8.5 g/L); Mg as magnesium sulfate MgSO_4_ (40.8 g/L); S as magnesium sulfate MgSO_4_ (54.4 g/L)	Single spraying BBCH 13-15 (LSS)	0.07%	300 l·ha^−1^/0.30 Mpa	June 21	June 20	June 7
		Single spraying BBCH 13-15 (HSS)	0.13%				
		Double spraying BBCH 13-15, BBCH 61 (LDS)	0.07%		June 21, July 5	June 20, July 3	June 7, June 23
		Double spraying BBCH 13-15, BBCH 61 (HDS)	0.13%				

Little data are provided in the available literature on the effect of biostimulants based on nitrophenols and titanium compounds on crops. In addition, we found no publications that would describe investigations on the effect of using both natural and synthetic biostimulants on the contents of dietary fiber and its fractions in the harvested crop.

This work presents a complex study addressing the action of the biostimulants Atonik and Tytanit and demonstrates their effect on the physiological traits, plant productivity, and seed yield quality of *Glycine max*, which is an extremely important crop from the economic standpoint.

The research hypothesis assumes that due to the various compositions, number of applications, and concentration of the biostimulants, they will induce different responses of plants.

In addition, considering that the mechanisms of action of the tested preparations have not been completely elucidated yet, though it was not the major objective of our study, we tried to hypothesize on the potential mechanisms of action of both the biostimulants to support the explanation of results obtained in our experiment.

## Materials and methods

### Plant materials and growth conditions

Study material originated from a field experiment conducted in the years 2014–2016 in Perespa village (50°66′N; 23°63′E, Poland), on soybean (*Glycine max* (L.) Merr.) of the Atlanta cultivar. The experiment was established in a randomized block design in four replications on experimental plots of area of 10 m^2^. Soybean was cultivated on the brown rendzina soil, characterized by alkaline pH (pH in 1M KCl:7.4–7.5). The contents of assimilable nutrients in the soil were at the medium levels: P (12.6–14.2 mg P_2_O_5_ in 100 g soil), K (15.3–17.1 mg K_2_O in 100 g soil), and Mg (6.2–6.8 mg Mg in 100 g soil). Each year, winter wheat was used as a forecrop. Soybean seeds were sown on the 25th of April in 2014, 25th of April in 2015, and 23rd of April in 2016 in rows every 30 cm at an approximate spacing of 3.5 cm. Weeds were removed mechanically and manually. In the growing season, the plants were sprayed with biostimulants according to the scheme of doses; developmental stages of plants and terms of spraying are presented in Table 1. Plants sprayed with water served as the control.

The Atlanta cultivar was selected for the study considering its phenotypic and functional traits. Its growing season ranges from 135 to 140 days and its plants reach the height of 100–130 cm. Its yielding potential exceeds 4 t/ha and its seeds are characterized by high protein (40–44%) and lipid (17.4–19%) contents. Its 1000-seed weight ranges from 180 to 185 g. These traits contribute to a high interest in its cultivation expressed by farmers and to its use in the feed and food industry.

The biostimulants were applied when foliar administration of microelement preparations is recommended. Their doses were adjusted based on recommendations for other crops, because there are no producer recommendations for doses in soybean cultivation.

The average temperature and rainfalls in the soybean growing season are shown in Table 2.

**Table 2:** Temperature (T) and rainfalls during the soybean growing season 2014–2016.

**Month**	**Year**						**Average from 2002 to 2013**	
	**2014**		**2015**		**2016**		
	**T (°C) Average (min/max)**	**Rainfall (mm)**	**T (°C) Average (min/max)**	**Rainfall (mm)**	**T (°C) Average (min/max))**	**Rainfall (mm)**	**T (°C)**	**Rainfall (mm)**
April	9.4 (−6.0/22.7)	36.5	8.2 (−1.7/24.3)	30.1	9.2 (−1.2/22.6)	68.4	8.5	41.2
May	13.7 (0.5/27.7)	208.3	12.7 (1.5/24.9)	108.6	13.8 (2.6/26.7)	61.3	12.7	63.4
June	16.1 (6.7/28.9)	67.1	17.4 (6.6/30.5)	14.1	18.1 (4.2/31.5)	97.1	17.7	68.6
July	20.3 (10.0/31.0)	104.2	19.6 (8.4/33.4)	59.2	19.5 (8.8/31.2)	107.6	18.9	79.1
August	18.2 (6.3/34.0)	115.4	21.6 (5.6/35.5)	23.4	18.2 (7.1/30.7)	95.3	19.4	71.8
September	13.7 (3.7/25.8)	89.4	15.1 (4.2/34.5)	137.6	15.2 (1.6/28.7)	41.2	14.1	69.2
Average/Total	15.1	620.9	15.8	373.0	17.1	470.9	15.2	393.3

### Plant yield and nutritional value determination

Determinations were conducted for plant height, internode number on the main shoot, first pod height, pod number per plant, seed number per 1 m^2^, seed weight, 1,000-seed weight, as well as the protein and lipid contents in the dry matter of seeds.

Protein content was determined by using the Kjeldahl method (AOAC, 2000, Official Method 992.23, 979.09), whereas content of lipids was determined based on the acid hydrolysis method (AOAC, 2000, Official Method 922.86).

### Nutraceutical potential

#### Phenolics content and antioxidant capacity determination

A seed extract was prepared following the methodology proposed by Swieca et al. (2012). Soybean seeds were ground and extracted with a mixture of acetone, water, and hydrochloric acid (70:29:1; v/v/v). Afterwards, the samples were centrifuged for 10 min (6,800 × g) and the resultant supernatant was collected and used for further analyses.

#### Phenolics determination

##### Determination of total phenolic compounds (TPC)

The content of total phenolic compounds (TPC) was determined with the method of Singleton and Rossi (1965) by using the Folin-Ciocalteau reagent. The absorbance of the samples was measured with a UV-vis spectrophotometer at the wavelength of 725 nm. TPC was computed and expressed as gallic acid equivalents (GAE) in mg per g of dry matter (DM).

##### Determination of flavonoid content (TFC)

The total content of flavonoids was determined according to the method presented by Lamaison and Carnet (1990). The prepared soybean extract was mixed with a methanolic solution of AlCl_3_ × 6H_2_O. After incubation, absorbance was measured with a UV-vis spectrophotometer at the wavelength of 430 nm. The total flavonoid content was expressed as quercetin equivalents (QE) in mg per g DM.

##### Determination of anthocyanins (TAC)

The content of anthocyanins was assayed with the method proposed by Fuleki and Francis (1968) using potassium chloride and sodium acetate buffer at two pH values (1.0 and 4.5). After 15 min, the absorbance of each sample was measured at the wavelengths of 520 and 700 nm. Then, the anthocyanin content was calculated as cynidin-3-glucoside equivalents (Cy3-GE) in mg per g DM.

#### Reducing power

Reducing power was measured by following the method provided by Pulido et al. (2000). The soybean extract was mixed with a phosphate buffer (200 mM, pH 6.6) and 1% solution of K_3_[Fe(CN_6_)]. Next, the samples were incubated at 50°C for 20 min. The reaction was stopped with trichloroacetic acid, and the samples were centrifuged (6,800 × g, 10 min). The resultant supernatant was mixed with distilled water and FeCl_3_. Then absorbance was measured at the wavelength of 700 nm. Reducing power was expressed as Trolox equivalents in mg per g DM.

#### Dietary fiber analysis

Determinations were conducted in three replications for the contents of neutral-detergent fiber (NDF) and acid-detergent fiber (ADF) fractions, and for the lignin (ADL) content in soybean samples according to the Van Soest et al. (1991) method using filtration bags and Ankom apparatus (Ankom220, USA). The NDF content was determined using a solution of neutral detergent (sodium-lauryl sulfate, ethylenediamine tetra acetic disodium salt, sodium borate, di-basic sodium phosphate, triethylene glycol), alpha-amylase (17,400 liquid units/mL, FAA Ankom Technology), and sodium sulfite (FSS Ankom Technology). The ADF content was determined using an acid detergent (trimethylammonium bromide, standardized sulfuric (VI) acid). Once the ADF content was determined, the lignin content was assayed in soybean samples using a standardized solution of sulfuric (VI) acid (Ankom Technology, FSA 72). The difference between the contents of NDF and ADF fractions was used to compute the hemicellulose (HCEL) content, and the difference between the contents of ADF and lignin (ADL) served to calculate the cellulose (CEL) content in soybean samples (Van Soest et al., 1991).

### The index of biostimulant effect

The index of biostimulant effect (ABT-C) was determined as the difference between the mean result obtained after biostimulant application (ABT) and control (C), enabling the evaluation of the effect of biostimulant type on the analyzed traits. The mean value for each treatment has been obtained by clustering the means in the cases of single spraying with the lower concentration (LSS), double spraying with the lower concentration (LDS), single spraying with the higher concentration (HSS), and double spraying with the higher concentration (HDS) from different years all together. The standard deviation (SD) value was determined for all reported mean values of ABT-C (Kocira et al., 2018a).

### Statistical analysis

The obtained results were statistically elaborated with Statistica 13 software (StatSoft, Inc.). The materials were collected over three seasons (2014–2016). The normality of data distribution was assessed with the Shapiro–Wilk test. The significance of differences between the evaluated mean values was estimated with the Tukey test at a significance level of *p* < 0.05. In each study season, samples were collected from the four plots for each combination, and laboratory analyses of each trait were carried out in three replications. To determine how strong the relationship between physiological effects and biochemical data was, the correlation coefficient was calculated.

## Results

### Effect of biostimulants on biometric traits

#### Plant height

A single application of the higher concentration (HSS) of Atonik ensured better effects in increasing soybean plant height (increase by 36% compared to the control) (Table 3). Similar dependencies were observed after the single and double use of Atonik in its lower concentration. However, the highest plants were obtained in the growing season 2014 after their double spraying with the lower concentration (LDS) of Tytanit. In contrast, the smallest plants were produced in the season 2015 and their height differed significantly from values noted in seasons 2014 and 2016 (Table 5). Both the tested biostimulants increased the height of plants, as indicated by the values of the index of biostimulant effect (ABT-C) ranging from 24.1 to 25.4 cm for this trait (Table 4).

**Table 3:** Effect of biostimulant treatment on biometric traits of soybean (average from 2014 to 2016).

**Parameters**	**Biostimulant treatment**	**Biostimulant**							
		**Atonik**				**Tytanit**			
		**Season**			**Average**	**Season**			**Average**
		**2014**	**2015**	**2016**		**2014**	**2015**	**2016**	
Plant height (cm)	C	85.4^a^	81.9^a^	88.1^a^	85.1^a^	85.4^a^	81.9^a^	88.1^a^	85.1^a^
	LSS	108.8^b^	97.4^b^	118.2^b^	114.9^b^	107.9^b^	101.8^b^	116.8^b^	108.8^b^
	LDS	110.3^b^	99.2^b^	117.8^b^	114.2^b^	120.0^b^	99.7^b^	114.6^b^	111.4^b^
	HSS	112.7^b^	99.9^b^	115.6^b^	115.8^b^	118.3^b^	97.5^b^	118.4^b^	111.4^b^
	HDS	114.5^b^	100.7^b^	115.3^b^	111.0^b^	113.7^b^	100.5^b^	170.0^b^	110.4^b^
	**Average**	**106.3^b^**	**95.8^a^**	**111.0^c^**		**109.1^b^**	**96.3^a^**	**111.0^b^**	
Number of internodes in the main shoot	C	11.2^a^	10.1^a^	9.6^a^	10.3^a^	11.2^ab^	10.1^a^	9.6^a^	10.3^bc^
	LSS	10.7^a^	10.6^a^	11.1^a^	13.0^a^	12.3^b^	10.5^a^	12.4^a^	11.7^c^
	LDS	10.5^a^	9.6^a^	11.4^a^	12.6^a^	7.2^a^	8.2^a^	8.5^a^	8.0^a^
	HSS	11.8^a^	10.9^a^	12.6^a^	14.0^a^	7.8^a^	8.4^a^	9.1^a^	8.4^ab^
	HDS	12.2^a^	9.8^a^	11.9^a^	11.3^a^	11.4^ab^	9.4^a^	10.0^a^	10.3^bc^
	**Average**	**11.3^b^**	**10.2^a^**	**11.3^b^**		**10.0^a^**	**9.3^a^**	**9.9^a^**	
Number of pods (per plant)	C	1793^a^	1581^a^	1907^a^	15.4^a^	15.2^a^	14.7^a^	16.3a	15.4a
	LSS	2238^b^	2428^bc^	2386^b^	21.4^c^	18.3^ab^	15.5^a^b	16.8a	16.9ab
	LDS	2235^b^	2576^c^	2529^b^	18.1^ab^	23.4^b^	19.5d	21.9b	21.6d
	HSS	2375^b^	2317^b^	2420^b^	17.7^ab^	18.7^ab^	19.0cd	16.8a	18.1bc
	HDS	2377^b^	2414^bc^	2445^b^	20.7^bc^	22.7^b^	17.4bc	19.8ab	20.0cd
	**Average**	**18.6^a^**	**19.7^a^**	**19.1^a^**		**19.7^c^**	**17.2^a^**	**18.3b**	
Location height of the first pod (cm)	C	12.5^a^	11.1^a^	11.7^a^	11.7^a^	12.5^a^	11.1^a^	11.7a	11.7a
	LSS	13.0^a^	14.6^b^	14.3^b^	13.4^a^	13.8^a^	12.5^a^	13.0a	13.1a
	LDS	13.5^a^	13.0^ab^	11.4^a^	12.1^a^	14.6^a^	13.5^a^	12.0a	13.4a
	HSS	13.8^a^	13.8^ab^	11.7^a^	13.0^a^	14.3^a^	13.3^a^	12.2a	13.3a
	HDS	12.5^a^	13.0^ab^	14.6^b^	13.3^a^	14.6^a^	13.0^a^	13.0a	13.5a
	**Average**	**13.1^a^**	**13.1^a^**	**12.7^a^**		**14.0^b^**	**12.7**^ab^	**12.4a**	
Number of seeds (per m^−2^)	C	1793^a^	1581^a^	1907^a^	1760^a^	1793^a^	1581^a^	1907^a^	1760^a^
	LSS	2238^b^	2428^bc^	2386^b^	2182^b^	2182^b^	2011^b^	2267^b^	2153^b^
	LDS	2235^b^	2576^c^	2529^b^	2270^bc^	2654^c^	2083^b^	2272^b^	2336^bc^
	HSS	2375^b^	2317^b^	2420^b^	2280^bc^	2468^c^	2141^b^	2476^bc^	2362^bc^
	HDS	2377^b^	2414^bc^	2445^b^	2420^c^	2692^c^	2122^b^	2595^c^	2470^c^
	**Average**	**2204^a^**	**2263**^ab^	**2338^b^**		**2358^b^**	**1988^a^**	**2303^b^**	
Seed yield (t ha^−1^)	C	3.267^a^	2.664^a^	3.262^a^	3.064^a^	3.267^a^	2.664^a^	3.262^a^	3.064^a^
	LSS	3.671^ab^	3.950^bc^	3.818^ab^	3.662^b^	3.581^a^	3.287^b^	3.685^bc^	3.518^b^
	LDS	3.691^ab^	4.208^c^	4.101^b^	3.806^bc^	4.448^c^	3.323^b^	3.590^ab^	3.787^bc^
	HSS	3.929^b^	3.702^b^	3.936^b^	3.715^bc^	4.018^b^	3.485^b^	4.065^cd^	3.856^bc^
	HDS	3.948^b^	3.930^bc^	4.059^b^	3.979^c^	4.446^c^	3.522^b^	4.322^d^	4.097^c^
	**Average**	**3.701^a^**	**3.691^a^**	**3.835^a^**		**3.952^c^**	**3.256^a^**	**3.785^b^**	
1000-seed weight (g 1000^−1^)	C	182.2^b^	168.5^a^	171.0^b^	173.9^b^	182.2^b^	168.5^b^	171.0^c^	173.9^b^
	LSS	164.0^a^	162.8^a^	160.2^a^	168.1^ab^	164.2^a^	163.4^ab^	162.6^ab^	163.4^a^
	LDS	165.2^a^	163.3^a^	162.1^a^	167.9^ab^	167.6^a^	159.7^a^	158.0^a^	161.8^a^
	HSS	165.7^a^	159.6^a^	162.5^a^	163.1^a^	163.0^a^	162.7^ab^	164.2^b^	163.3^a^
	HDS	166.0^a^	162.8^a^	166.0^ab^	164.4^a^	165.3^a^	166.0^ab^	167.0^bc^	165.9^a^
	**Average**	**168.6^b^**	**163.4^a^**	**164.4^a^**		**168.4^b^**	**164.1^a^**	**164.5^a^**	

*C, control; LSS, lower concentration single spraying; LDS, lower concentration double spraying; HSS, higher concentration single spraying; HDS, higher concentration double spraying. Means in the columns, concerning the selected traits, followed by different small letters are significantly different at p < 0.05*.

**Table 4:** The index of biostimulant effect (ABT-C).

**Parameters**	**Preparat**							
	**Atonik**				**Tytanit**			
	**2014**	**2015**	**2016**	**Average**	**2014**	**2015**	**2016**	**Average**
Plant height (cm)	26.2^b^ (±2.5)	17.4^a^ (±1.4)	28.7^b^ (±1.5)	24.1a (±5.3)	29.6^b^ (±5.4)	18.0^a^ (±1.8)	28.6^b^ (±1.6)	25.4^a^ (±6.3)
Number of internodes in the main shoot	0.1^a^ (±0.9)	0.1^a^ (±0.6)	2.2^b^ (±0.6)	0.8^b^ (±1.2)	−1.5^a^ (±2.5)	−1.0^a^ (±1.1)	0.4^a^ (±1.7)	−0.7^a^ (±2.0)
Location height of the first pod (cm)	0.7^a^ (±0.6)	2.5^a^ (±0.8)	1.3^a^ (±1.7)	1.5^a^ (±1.3)	1.9^b^ (±0.4)	2.0^b^ (±0.4)	0.9^a^ (±0.5)	1.6^a^ (±0.7)
Number of pods (per plant)	4.2^a^ (±0.9)	6.2^a^ (±3.0)	3.5^a^ (±1.5)	4.6^a^ (±2.2	5.6^a^ (±2.7)	3.2^a^ (±1.8)	2.6^a^ (±2.5)	3.8^a^ (±2.5)
Number of seeds (per m^−2^)	513.3^a^ (±80.4)	853.3^b^ (±106.7)	538.4^a^ (±60.8)	635.0^a^ (±178.9)	705.8^a^ (±232.9	508.9^a^ (±57.4)	495.6^a^ (±161.5)	570.1^a^ (±181.3)
Seed yield (t ha^−1^)	0.543^a^ (±0.150)	1.283^b^ (±0.207)	0.716^a^ (±0.128)	0.847^a^ (±0.362)	0.857 (±0.414)	0.740^a^ (±0.116)	0.654^a^ (±0.340)	0.750^a^ (±0.299)
1000-seed weight (g 1000^−1^)	−17.0^a^ (±0.9)	−6.4^b^ (±1.7)	−8.3^b^ (±2.4)	−10.6^a^ (±5.1)	−17.2^a^ (±2.0)	−5.5^b^ (±2.6)	−8.2^b^ (±3.6)	−10.3^a^ (±5.8)
Total protein (% DM)	−2.7^a^ (±0.8)	−1.0^a^ (±3.1)	−2.2^a^ (±1.0)	−2.0^a^ (±1.9)	−0.7^a^ (±1.2)	−1.8^a^ (±1.5)	−0.5^a^ (±0.9)	−1.0^a^ (±1.3)
Total fat (% DM)	−2.5^a^ (±0.8)	0.0^b^ (±1.7)	−1.9^ab^ (±0.5)	−1.5^a^ (±1.5)	−2.3^a^ (±0.3)	−0.9^b^ (±0.4)	−2.0^a^ (±0.4)	−1.7^a^ (±0.7)

*Values followed by different small letters are significantly different at p < 0.05*.

**Table 5:** Effect of seasons on biometric traits, soybean yield, and nutritional properties.

**Parameters**	**Season**		
	**2014**	**2015**	**2016**
Plant height (cm)	107.9^b^ (±2.7)	96.0^a^ (±2.2)	111.0^c^ (±1.4)
Number of nodes in the main shoot	10.6^a^ (±1.1)	9.7^a^ (±0.6)	10.7^a^ (±1.1)
Location height of the first pod (cm)	13.5^b^ (±0.9)	12.9^ab^ (±0.8)	12.6^a^ (±0.4)
Number of pods (per plant)	19.1^a^ (±0.9)	18.4^a^ (±1.4)	18.7^a^ (±0.6)
Number of seeds (per m^−2^)	2280.6^b^ (±89.9)	2125.4^a^ (±152.1)	2320.4^b^ (±66.2)
Seed yield (t ha^−1^)	3.827^b^ (±0.146)	3.474^a^ (±0.240)	3.810^b^ (±0.136)
1000-seed weight (g 1000^−1^)	168.5^b^ (±1.3)	168.7^a^ (±1.5)	164.4^a^ (±1.5)
Total protein (% DM)	35.5^a^ (±0.9)	45.4^b^ (±0.6)	34.8^a^ (±0.8)
Total fat (% DM)	15.6^b^(±0.1)	14.7^a^ (±0.4)	15.0^a^ (±0.1)

*Values followed by different small letters are significantly different at p < 0.05*.

#### Number of internodes in the main shoot

The highest number of internodes was observed after a single application of the higher concentration of the Atonik preparation (increase by 36% compared to the control) (Table 3). In the case of Tytanit, the value of this trait was increased only after single spraying with the lower concentration (LSS). The highest number of internodes on the main shoot was obtained in the first season and it differed significantly from the number determined in 2015 (Table 5). The values of the ABT-C index computed for Atonik were positive, whereas those calculated for Tytanit were negative (Table 4).

#### Height of the first pod location

Each of the applied biostimulants increased the height of the first pod location compared to the control, but results achieved with biostimulants did not differ significantly from those obtained in the control combination (Table 3). The highest values were obtained after a single application of the lower concentration of Atonik and a double application of the lower concentration of Tytanit. The greatest heights of the first pod locations on plants were observed in season 2014; however, they did not differ significantly from the values reported in the other two seasons (Table 5). The values of the ABT-C index computed for Atonik and Tytanit were positive and at a similar level (Table 4).

#### Number of pods per plant

A double foliar application of the lower concentration of Tytanit allowed for the achievement of the highest number of pods per plant (increase by 40%, respectively, compared to the control) (Table 3). When treating soybean plants with Atonik, the highest pod number was obtained after a single application of its lower concentration. The study demonstrated that the mean number of pods determined in particular growing seasons was at a similar level and did not differ significantly among seasons. Biostimulants increased the pod number per plant and the values of the index of the biostimulant effect ranged from 3.8 pods/plant after spraying with Tytanit to 4.6 pods/plant after spraying with Atonik (Table 4). However, no significant differences were found between values of this index determined for particular biostimulants.

### Effect of biostimulants on soybean yield

#### Number of seeds

Double spraying of soybean plants with the higher concentrations (HDS) of the tested biostimulants had the greatest effect on the increase in the seed number per m^2^. The highest seed number was achieved after Tytanit application (increase by 40% compared to the control) (Table 3). The analysis of growing seasons demonstrated the highest value of this trait in 2016, and the lowest one in 2015 (lower by 9% than that noted in 2016) (Table 5). The application of each stimulant increased this number, as indicated by the values of the ABT-C index calculated for this trait (Table 4). In turn, no significant differences were observed in the value of this index between the tested biostimulants; however, the most positive effect was observed upon the use of Atonik.

#### Seed yield

The most positive response of plants to the use of biostimulants was observed after their double spraying with the higher concentration of Tytanit, as indicated by their seed yield increase by 33% compared to the control. In this case, the seed yield exceeded 4 t/ha. The increase in seed yield was also observed upon double spraying the plants with the higher concentration of Atonik (Table 3). Seed yield below 4 t/ha was obtained only after the application of Atonik, but still it was higher by 20–30% than in the control combination. The highest mean seed yield for Atlanta cv. was obtained in 2016. In contrast, the seed yield of the 2015 season turned out to be the lowest among the studied seasons and differed significantly from the mean yields reported in 2014 and 2016 (Table 5). The foliar application of the tested biostimulants increased the seed yield of Atlanta cv. soybeans, as indicated by the positive values of the ABT-C index calculated for this trait (Table 4).

#### 1000-seed weight

The foliar application of the analyzed biostimulants decreased the 1000-seed weight. Its lowest value was determined after a double application of Tytanit in the lower concentration (decrease by 7.5% compared to the control) (Table 3). The least decrease of the 1000-seed weight was achieved after single plant spraying with the lower concentration of Atonik. The highest mean 1000-seed weight was reported in the growing season 2014. The values of the biostimulant effect index calculated for this trait were negative, pointing to the negative impact of the tested preparations on the 1000-seed weight.

### Effect of biostimulant on the nutritional properties of soybean seeds

#### Total protein in soybean seeds

**Figure 1:**
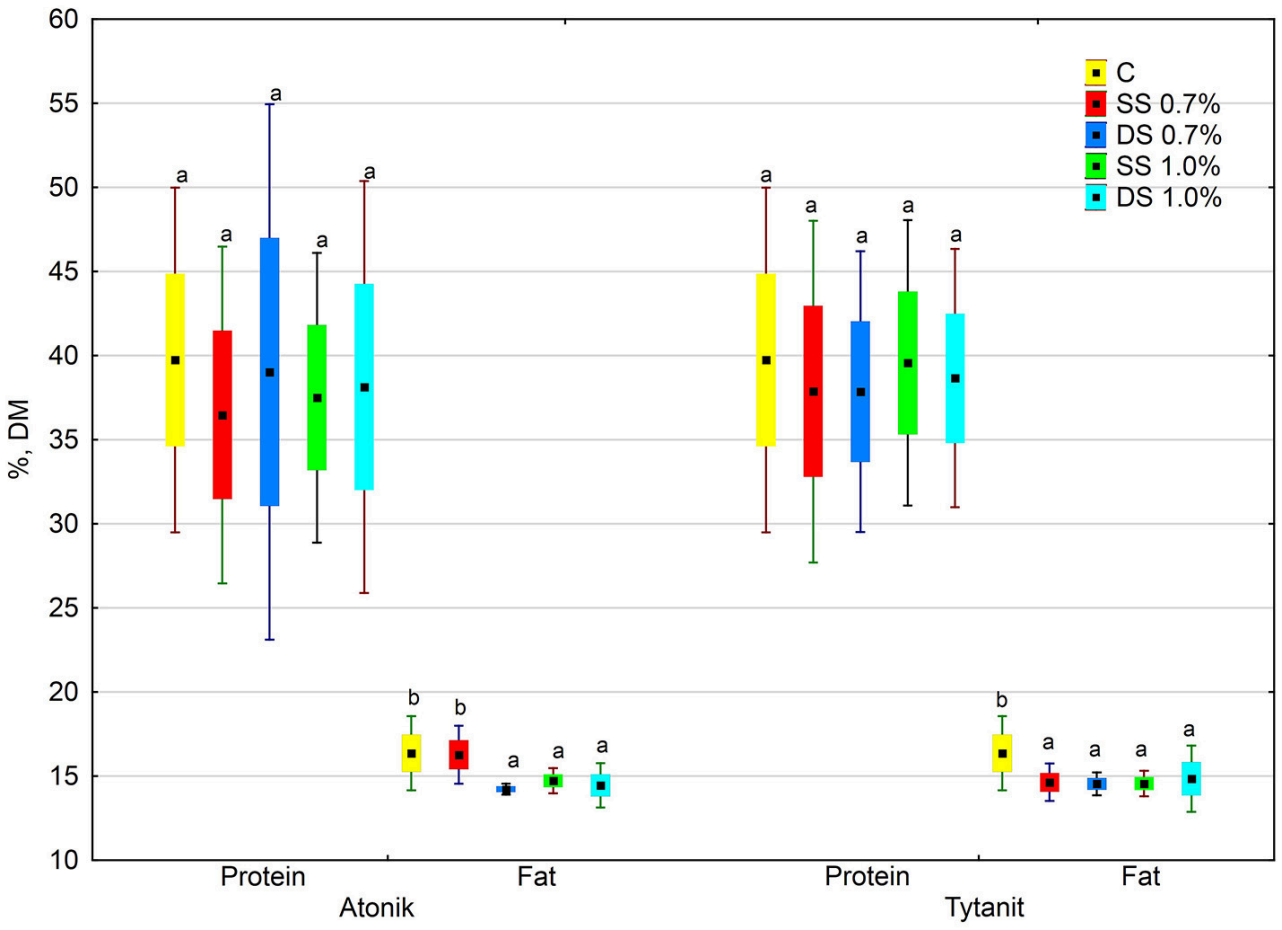
Effect of biostimulant treatment on protein and fat (average from 2014 to 2016). Values followed by different small letters are significantly different at *p* < 0.05.

**Figure 2:**
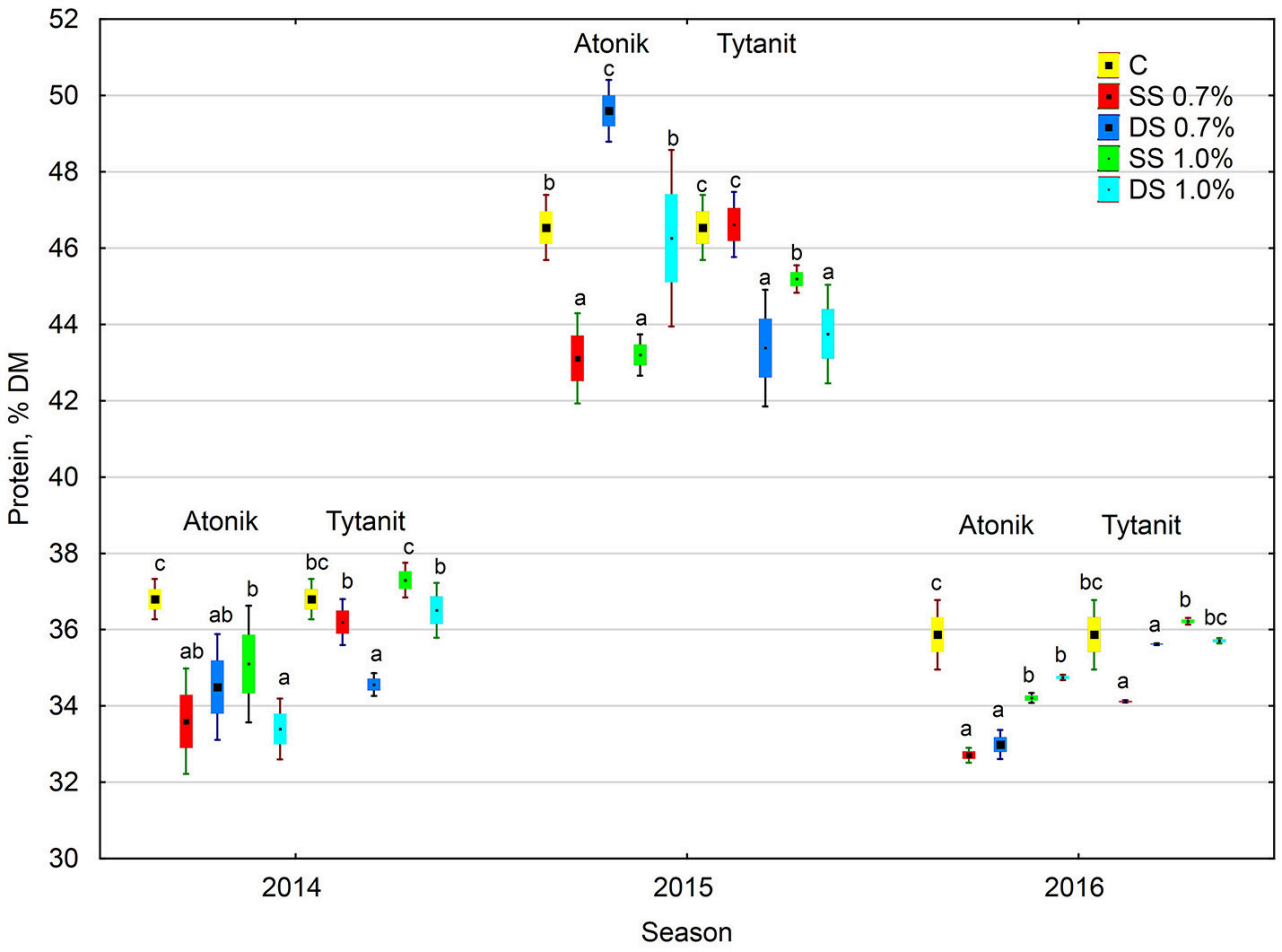
Effect of biostimulant treatment on protein content in soybeans in 2014–2016. Values followed by different small letters are significantly different at *p* < 0.05.

The protein content in the dry matter of seeds from the plants treated with the tested biostimulants varied. Depending on the concentration and number of applications, particular biostimulants either slightly increased or decreased its value (Figures 1, 2). Single spraying with the higher concentration (HSS) of the Tytanit preparation increased the protein content in soybean seeds only to a small extent. In turn, the use of Atonik decreased the protein content value regardless of the number of applications and concentration of the biostimulant. Considering the growing seasons, the highest protein content of seeds was noted in 2015 (Table 5, Figure 2). In addition, the values of the ABT-C index calculated for this trait were negative for both Atonik and Tytanit (Table 4).

#### Total fat in soybean seeds

**Figure 3:**
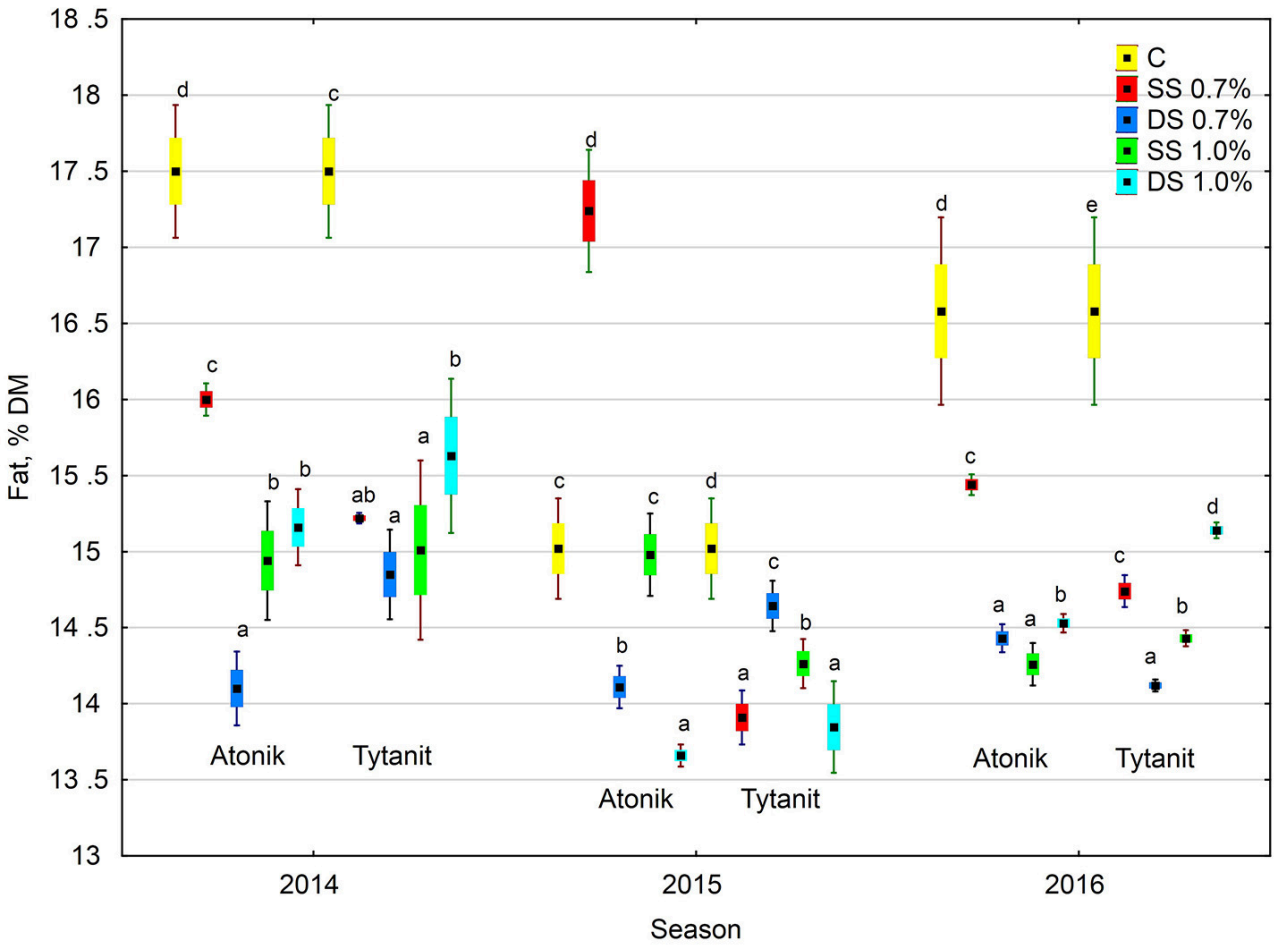
Effect of biostimulant treatment on fat content in soybeans in 2014–2016. Values followed by different small letters are significantly different at *p* < 0.05.

Regardless of the number of sprayings and concentration of biostimulants, their use decreased the fat content in the dry matter of soybean seeds, with the greatest reduction (by 15.5% compared to the control) noted after double spraying the plants with the lower concentration of Atonik (Figures 1, 3). In contrast, the least decrease in the fat content of the seeds compared to the control was determined after single spraying with the lower concentrations of Atonik and after double spraying with the higher concentrations of Tytanit. The highest fat content of soybean seeds was noted in season 2014, and a similar fat content was observed in 2016 (Table 5, Figure 3). The lowest fat content of the seeds was demonstrated in 2015. The values of the ABT-C index calculated for both the biostimulants were negative (Table 4), indicating their negative effect on the fat content of Atlanta cv. soybean seeds.

### Effect of biostimulants on the antioxidant potential in soybean seeds

#### Total phenolic content

The use of two different biostimulants in soybean cultivation caused changes in the total polyphenol content (TPC) in seeds (Table 6) that varied depending on both the number of applications and concentration of the tested preparations. In the case of plants sprayed with Atonik based on phenolics compounds, the TPC content increased compared to the control seeds. The highest content of total polyphenols in soybean seeds was determined after a single application of this biostimulant in its higher dose.

**Table 6:** Effect of biostimulant treatment on the antioxidant potential of soybean seeds.

**Parameters**	**Biostimulant treatment**	**Biostimulant**							
		**Atonik**				**Tytanit**			
		**Season**			**Average**	**Season**			**Average**
		**2014**	**2015**	**2016**		**2014**	**2015**	**2016**	
Total phenols (mg g^−1^ DM)	C	5.77^a^	4.50^a^	5.77^a^	5.35^a^	5.77^a^	4.50^a^	5.77^a^	5.35^a^
	LSS	8.82^a^	5.86^c^	5.93^b^	6.87^ab^	7.65^e^	7.35^c^	7.63^e^	7.55^c^
	LDS	5.69^a^	4.40^a^	5.84^a^	5.31^a^	7.12^c^	7.78^d^	7.07^c^	7.32^bc^
	HSS	10.02^a^	5.35^b^	11.24^d^	8.87^b^	6.94^b^	8.03^e^	6.80^b^	7.26^bc^
	HDS	7.11^a^	6.36^d^	7.29^c^	6.92^ab^	7.23^d^	5.06^b^	7.47^d^	6.59^b^
	**Average**	**7.48^b^**	**5.29^a^**	**7.21^b^**		**6.94^b^**	**6.55^a^**	**6.95^b^**	
Total flavonoids (mg g^−1^ DM)	C	1.99^a^	1.44^b^	1.99^a^	1.81^a^	1.99^c^	1.44^b^	1.99^c^	1.81^ab^
	LSS	2.59^c^	3.28^d^	2.68^c^	2.85^c^	2.15^d^	2.89^d^	2.19^d^	2.41^b^
	LDS	3.00^d^	1.29^a^	3.01^d^	2.43^bc^	1.30^a^	2.65^c^	1.36^ab^	1.77^a^
	HSS	3.66^e^	3.71^e^	3.65^e^	3.67^d^	1.32^a^	2.64^c^	1.31^a^	1.76^a^
	HDS	2.16^b^	1.63^c^	2.13^b^	1.97^ab^	1.45^b^	1.36^a^	1.41^b^	1.41^a^
	**Average**	**2.68^b^**	**2.27^a^**	**2.69^b^**		**1.64^a^**	**2.20^b^**	**1.65^a^**	
Anthocyanin (mg g^−1^ DM)	C	0.00^a^	0.00^a^	0.00^a^	0.000^a^	0.00^a^	0.00^a^	0.00^a^	0.000^a^
	LSS	0.00^a^	0.00^a^	0.00^a^	0.000^a^	0.00^a^	0.00^a^	0.00^a^	0.000^a^
	LDS	0.00^a^	0.02^c^	0.00^a^	0.006^ab^	0.08^b^	0.03^c^	0.08^b^	0.063^b^
	HSS	0.01^b^	0.01^b^	0.01^b^	0.010^b^	0.00^a^	0.01^b^	0.00^a^	0.003^a^
	HDS	0.01^b^	0.01^b^	0.02^c^	0.013^b^	0.00^a^	0.00^a^	0.00^a^	0.000^a^
	**Average**	**0.004^a^**	**0.008^c^**	**0.006^b^**		**0.016^b^**	**0.008^a^**	**0.016^b^**	
Reducing power (mg TE g^−1^DM)	C	0.15^a^	0.10^c^	0.15^a^	0.13^a^	0.15^a^	0.10^ab^	0.15^a^	0.13^a^
	LSS	0.15^a^	0.06^a^	0.17^b^	0.13^a^	0.19^b^	0.10^ab^	0.42^e^	0.31^b^
	LDS	0.19^b^	0.08^b^	0.21^c^	0.16^a^	0.41^d^	0.08^a^	0.22^b^	0.16^a^
	HSS	0.37^c^	0.10^c^	0.36^d^	0.28^ab^	0.27^c^	0.10^ab^	0.26^c^	0.21^ab^
	HDS	0.54^d^	0.06^a^	0.52^e^	0.37^b^	0.28^c^	0.12^b^	0.29^d^	0.23^ab^
	**Average**	**0.28^b^**	**0.08^a^**	**0.28^b^**		**0.26^b^**	**0.10^a^**	**0.27^b^**	

*C, control; LSS, lower concentration single spraying; LDS, lower concentration double spraying; HSS, higher concentration single spraying; HDS, higher concentration double spraying. Means in the columns, concerning the selected traits, followed by different small letters are significantly different at p < 0.05*.

The content of phenolics in soybean seeds increased compared to the control seeds upon the use of Tytanit, even in its lower concentration. Soybean responded with the greatest increase in the seed TPC content after a single spray with 0.07% Tytanit (increase by over 40% compared to the control).

**Figure 4:**
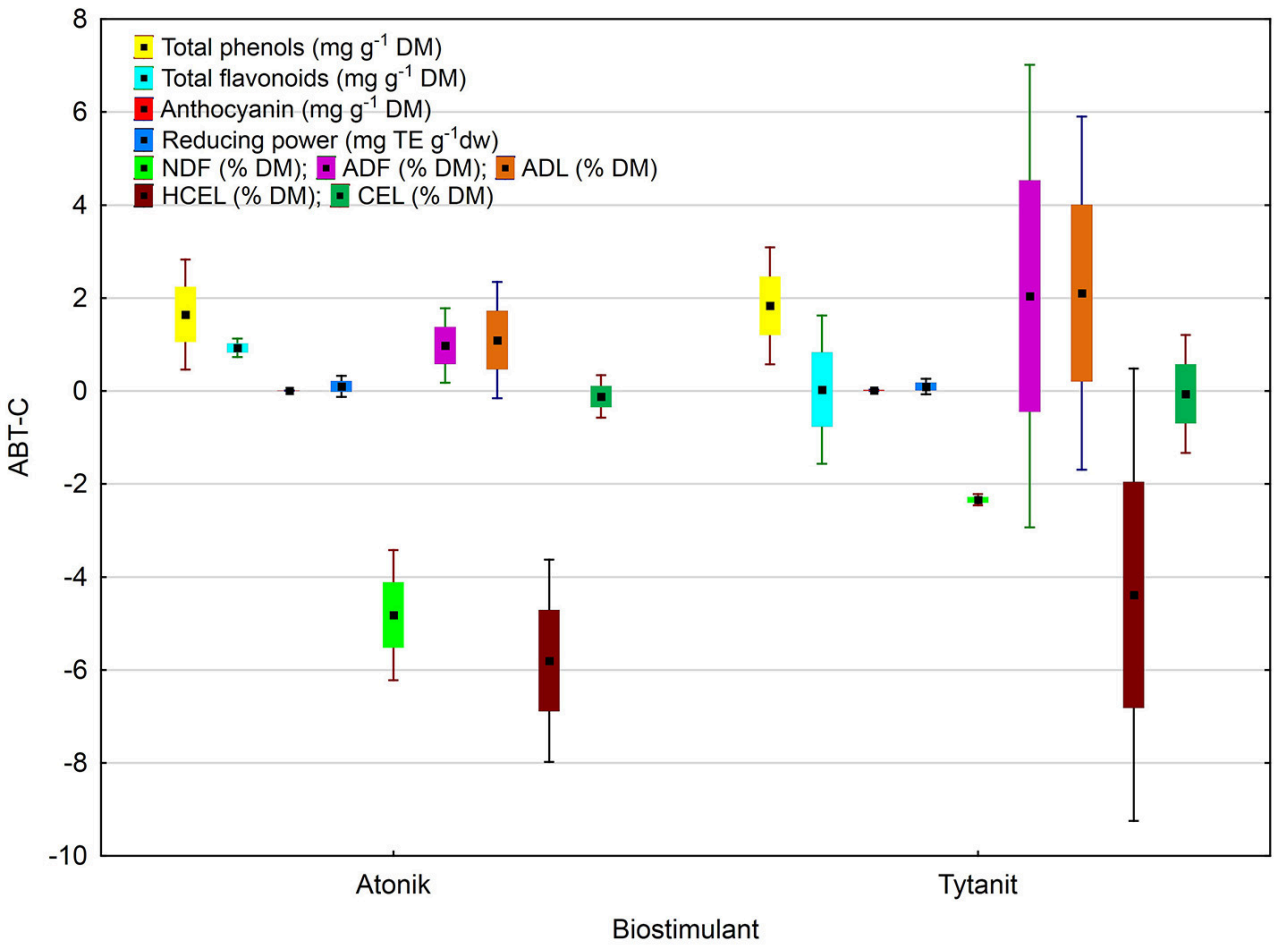
Antioxidant potential and fiber fractions of soybean seeds: the index of biostimulant effect. Values followed by different small letters are significantly different at *p* < 0.05.

**Figure 5:**
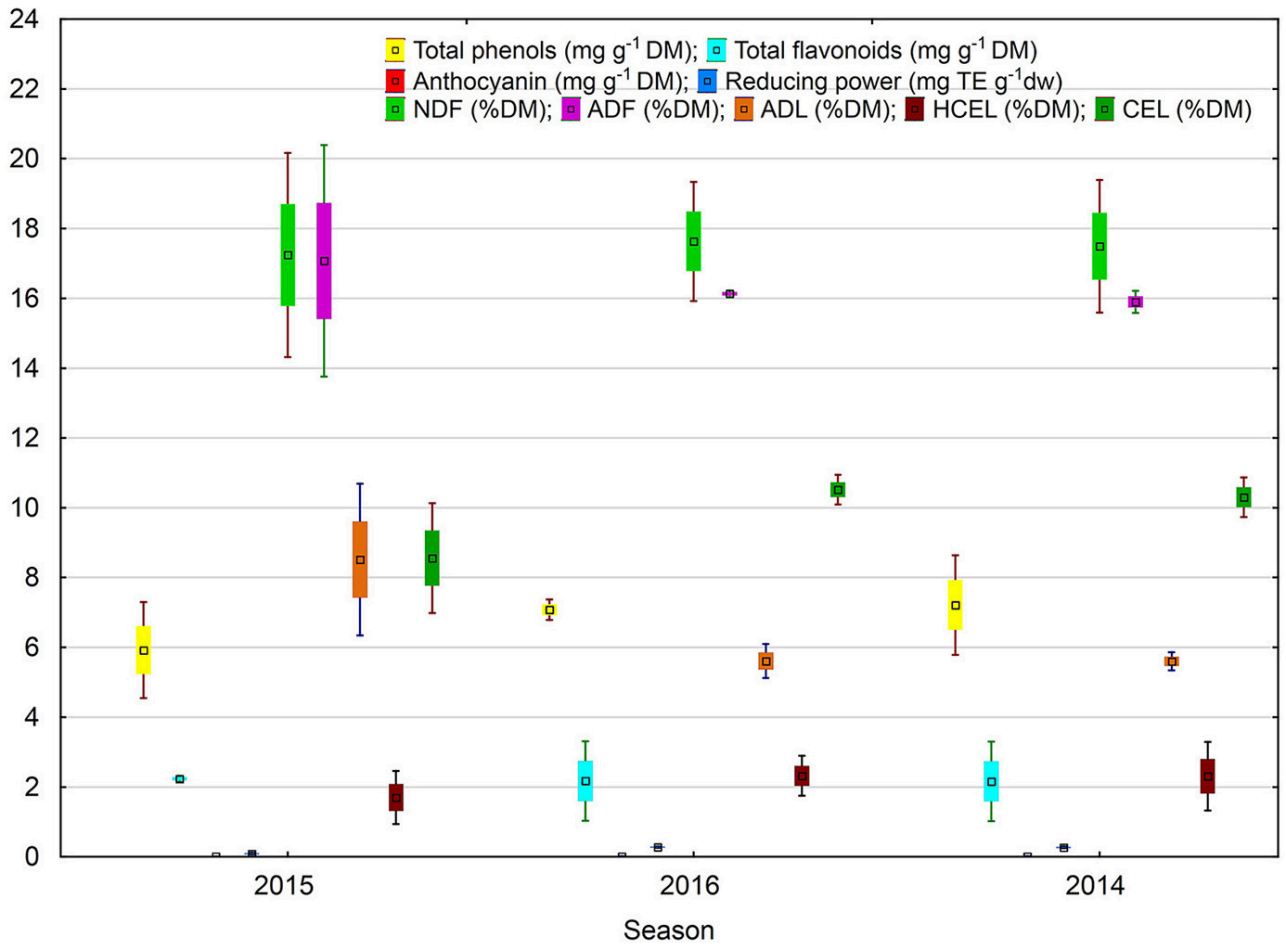
Effect of season on antioxidant potential and fiber fractions of soybean seeds. Values followed by different small letters are significantly different at *p* < 0.05.

A complex analysis of the average effect of biostimulants on the total phenolics content demonstrated the highest TPC after the application of the Tytanit preparation. A positive value of the difference between the polyphenol content in the combinations treated with biostimulants and control samples (ABT-C) was determined for all analyzed soybean seeds (Figures 4, 5). However, the statistical analysis indicated that the differences between the effects of these preparations on TPC were insignificant.

#### Total anthocyanin content

The presence of anthocyanins was detected in 11 out of the 30 analyzed combinations of the two biostimulants used in soybean cultivation. These compounds were not detected in control samples in any of the growing seasons studied.

The application of Atonik in its higher concentration led to an increase in the content of anthocyanins. Its highest value was determined in seeds from plants double-sprayed with the preparation based on phenolic compounds.

The content of anthocyanins in soybean seeds increased compared to that in control seeds after a double application of the higher concentration of Tytanit. The presence of anthocyanin contents was detected in seeds from only four out of the 16 tested combinations. The highest average content of anthocyanins was determined in seeds of soybeans double-sprayed with 0.07% Tytanit.

#### Total flavonoid content

Flavonoid content analysis showed a significant effect of the biostimulants on the values of flavonoid content. The treatment of plants with the Atonik preparation caused an increase in the total content of flavonoids in soybean seeds. Their highest content was determined after single spraying of the plants with the higher concentration of this preparation (over two-fold increase compared to the control). The content of flavonoids tended to increase also after spraying the plant with the lower concentration of this preparation.

Different observations were made after the foliar application of Tytanit. Only single spraying with its lower concentration caused an increase in the total flavonoid content, whereas its higher dose decreased the value of this trait.

The analysis of the effects of biostimulants having different compositions revealed that their foliar application resulted in an increased content of flavonoids compared to the control samples (a positive value of the ABT-C difference). However, no significant differences were found in the effects of these preparations.

#### Reducing power

The evaluation of the effect of applying biostimulants having different compositions on the antioxidant activity of soybean included the determination of reducing power. Its value was increased by almost all combinations of the tested biostimulants.

In the case of Atonik application, the highest value of reducing power was noted after double spraying the plants with its 0.2% solution that caused an almost 3-fold increase in the value of this trait compared to the control. In turn, single spraying with the same dose of this preparation caused almost a 2-fold difference in reducing power compared to the control combination. Significant differences in the values of reducing power were also noted after the use of Tytanit. The highest value of this trait was noted after a single application of the 0.07% biostimulant solution (increase by over 130% compared to the control samples).

### Effect of biostimulants on the fiber in soybean seeds

#### Neutral-detergent fiber

The content of the NDF in soybean seeds decreased compared to that in the control seeds after the application of both biostimulants. In none of the combinations did the NDF content exceed the value noted in the untreated samples (Table 7).

**Table 7:** Effect of biostimulant treatment on the fiber fractions in soybean seeds.

**Parameters**	**Biostimulant treatment**	**Biostimulant**							
		**Atonik**				**Tytanit**			
		**Season**			**Average**	**Season**			**Average**
		**2014**	**2015**	**2016**		**2014**	**2015**	**2016**	
NDF (% DM)	C	20.34^e^	20.40^e^	20.28^c^	20.34^b^	20.34^e^	20.40^e^	20.28^d^	20.34^c^
	LSS	12.85^a^	9.57^a^	12.91^a^	11.78^a^	16.69^b^	19.00^d^	16.65^b^	17.45^ab^
	LDS	12.96^b^	16.26^c^	13.00^a^	14.07^a^	19.34^c^	17.60^b^	19.29^c^	18.74^bc^
	HSS	17.23^c^	20.16*s*	17.78^b^	18.39^b^	20.04^d^	17.21^a^	20.71^e^	19.32^c^
	HDS	19.80^d^	13.16^b^	20.30^c^	17.75^b^	15.46^a^	18.69^c^	15.10^a^	16.41^a^
	**Average**	**16.64^b^**	**15.91^a^**	**16.85^c^**		**18.37^a^**	**18.58^b^**	**18.58^a^**	
ADF (% DM)	C	15.51^b^	14.54^b^	15.47^b^	15.17^a^	15.51^a^	14.54^a^	15.47^a^	15.17^a^
	LSS	16.23^d^	12.96^a^	16.45^d^	15.21^a^	16.25^b^	20.05^b^	16.86^c^	17.72^a^
	LDS	15.99^c^	18.46^e^	15.94^c^	16.80^b^	15.47^a^	19.24^b^	16.07^b^	16.93^a^
	HSS	14.62^a^	15.84^c^	15.06^a^	15.17^a^	15.36^a^	19.72^b^	15.85^b^	16.98^a^
	HDS	17.91^e^	16.64^d^	17.54^e^	17.36^b^	16.24^b^	18.76^b^	16.58^c^	17.19^a^
	**Average**	**16.05^b^**	**15.69^a^**	**16.09^c^**		**15.77^a^**	**18.46^b^**	**16.174^a^**	
ADL (% DM)	C	4.89^b^	6.07^a^	4.91^b^	5.29^ab^	4.89^a^	6.07^a^	4.91^a^	5.29^a^
	LSS	7.01^e^	6.26^b^	6.92^e^	6.73^ab^	7.15^e^	9.35^b^	7.11^b^	7.87^a^
	LDS	5.20^c^	10.60^e^	5.57^c^	7.12^b^	5.02^b^	11.61^e^	4.89^a^	7.17^a^
	HSS	4.33^a^	6.64^c^	4.32^a^	5.10^a^	5.18^c^	10.00^c^	5.14^ab^	6.77^a^
	HDS	5.96^d^	8.06^d^	5.81^d^	6.61^ab^	6.32^d^	10.51^d^	6.52^ab^	7.78^a^
	**Average**	**5.48^a^**	**7.53^c^**	**5.51^b^**		**5.71^a^**	**9.51^b^**	**5.71^a^**	
HCEL (% DM)	C	4.83^d^	5.86^c^	4.81^c^	5.17^d^	4.83^d^	5.86^c^	4.81^c^	5.17^c^
	LSS	0.00^a^	0.00^a^	0.00^a^	0.00^a^	0.44^b^	0.00^a^	0.00^a^	0.15^a^
	LDS	0.00^a^	0.00^a^	0.00^a^	0.00^a^	3.87^c^	0.95^a^	3.22^b^	2.68^b^
	HSS	2.61^c^	4.32^b^	2.72^b^	3.21^c^	4.68^d^	0.00^a^	4.86^b^	3.18^b^
	HDS	1.89^b^	0.00^a^	2.76^b^	1.55^b^	0.00^a^	0.00^a^	0.00^a^	0.00^a^
	**Average**	**1.87^a^**	**1.04^b^**	**2.06^b^**		**2.76^b^**	**1.36^a^**	**2.58^b^**	
CE (% DM)	C	10.52^c^	8.47^c^	10.56^c^	9.85^ab^	10.52^d^	8.47^a^	10.56^a^	9.85^a^
	LSS	9.22^a^	6.70^a^	9.53^a^	8.48^a^	9.10^a^	10.70^a^	9.75^a^	9.85^a^
	LDS	10.29^b^	7.86^b^	10.37^b^	9.67^ab^	10.45^d^	7.63^a^	11.18^a^	9.75^a^
	HSS	10.79^d^	9.20^d^	10.74^d^	10.08^ab^	10.18^c^	9.72^a^	10.71^a^	10.20^a^
	HDS	11.95^e^	8.58^c^	11.73^e^	10.75^b^	9.92^b^	8.25^a^	10.06^a^	9.41^a^
	**Average**	**10.55^b^**	**8.16^a^**	**10.58^b^**		**10.03^a^**	**8.95^a^**	**10.45^a^**	

*C, control; LSS, lower concentration single spraying; LDS, lower concentration double spraying; HSS, higher concentration single spraying; HDS, higher concentration double spraying. Means in the columns, concerning the selected traits, followed by different small letters are significantly different at p < 0.05*.

There were no significant differences in the NDF content between control samples and the samples treated with the higher concentration (0.2%) of Atonik preparation. A significant decrease in the NDF content was determined after the foliar application of 0.1% Atonik solution.

In the case of the Tytanit preparation, the NDF content determined after a single application of its lower concentrations was similar to the value noted for the control sample. In the other analyzed treatment combinations, NDF contents were lower.

The negative value of the difference between the NDF contents in seeds of plants treated with the two biostimulants and in control samples (ABT-C index) was noted for all analyzed soybean combinations (Figure 4). However, the highest value was observed for the soybean plants treated with Atonik. The value of the analyzed index proves no significant differences in the response of soybean plants to the treatment with biostimulants.

#### Acid-detergent fiber

An analysis of the ADF content showed that it depended on the type of biostimulant used for the spraying of plants. An application of Atonik caused an increase in the ADF content compared to the control. The greatest significant increase was observed after the double application Atonik in both its concentrations.

The foliar application of Tytanit resulted in an increased ADF content. The conducted statistical analyses demonstrated that soybean did not respond to the application of Tytanit with a significant change in the ADF content.

#### Lignin

In the case of Atonik preparation, only double spraying of plants with its concentration of 0.1% caused a significant increase in the ADL content compared to the other combinations. In turn, a lower ADL content was observed after the double treatment of soybean with 0.2% Atonik solution (decrease by 3.5% compared to the control).

The use of Tytanit caused an increase in the ADL content compared to the control seeds. As in the case of ADF analysis, soybean plants did not respond to Tytanit treatment with a change in the ADL content.

#### Hemicellulose and cellulose

Cellulose, hemicellulose, and lignin are the major fractions of crude fiber, while hemicelluloses (HCEL) are the key components of dietary fiber in legume grains. The contents of these fibers can be computed from a difference between the contents of the neutral fraction (NDF) and the acid fraction (ADF) of dietary fiber. The use of biostimulants with different compositions contributed to a decrease in the HCEL content in soybean seeds compared to the control sample.

In turn, the difference between the contents of the acid fraction (ADF) and the lignin fraction (ADL) of dietary fiber provides information on the content of cellulose (CEL). The application of biostimulants differentiated the CEL contents in soybean seeds. Double spraying of plants with a higher concentration of Atonik resulted in the highest content of cellulose in soybean seeds (increase by 9% compared to the control), whereas a foliar application of Tytanit caused no changes in the cellulose content compared to the control sample.

The results of dietary fiber fraction analysis demonstrated that in the case of ADF and ADL contents analyses showed a positive, while in the case of NDF and HCEL contents a negative, value of the difference between their contents in combinations treated with biostimulants and the control samples (ABT-C). Significant differences were observed only in the case of CEL contents, owing to the effects exhibited by the tested preparations in soybean cultivation (Figure 4).

## Discussion

The analysis of the results obtained in our study demonstrated that plant growth, as well as yield and biometric traits of soybean seeds, depended on the type of biostimulants, their concentration, and number of their applications. The physiological response of soybean plants to these preparations was mainly due to their so-called active (activating) compounds, such as phenols (Jindo et al., 2012; Ertani et al., 2013, 2014; Swieca et al., 2014; Swieca, 2016), which are active substances of the Atonik preparation. However, as reported by Ertani et al. (2011) and Kauffman et al. (2007), the highest effectiveness of biostimulants in crop cultivation is achieved upon the use of their minimal doses, although these authors emphasize that the effects of the preparations depend, most of all, on plant species and cultivar and on plant growth stage. The investigations conducted by Azcona et al. (2011) and Ertani et al. (2012, 2014) have also proved that the differences in the effects of biostimulants are due to the number of treatments performed at the appropriate BBCH stages. The first treatment of plants with these preparations results mainly in the increased number and weight of leaves, and is referred to as the short-time effect. A subsequent dose of biostimulants, applied at the blooming stage of plants, leads to a long-term effect that is manifested by changes in crop productivity and yield quality (Nardi et al., 2009; Ertani et al., 2014). A distinct increase observed in our study in the number of pods and seeds, in seed yield, and in the antioxidative activity of soybean seeds may be due to the use of biostimulants at the appropriate stages of plant growth. The experiments carried out by Oboh et al. (2007) and by Zhang and Hamauzu (2003) confirmed that the first application of biostimulants led to an increased content of phenolic acids in leaves, whereas the second application caused a lesser increase in their content. Zarzecka et al. (2017) demonstrated that the use of a biostimulant based on phenolic compounds (Atonik) resulted in an increased yield and accumulation of phenolic acids in potato tubers. Our study demonstrated also that the use of synthetic biostimulants determined the protein and fat contents in soybean seeds that tended to decrease regardless of the number of treatments or concentrations of the tested preparations. This was also confirmed in our previous experiment (Kocira et al., 2017a), in which Atonik application led to a decrease in the protein and fat contents in common bean seeds.

The results of our previous studies confirmed that the use of different doses and concentrations of Tytanit and Atonik resulted not only in the increased content of polyphenolic compounds but also in the increased antioxidative potential of common bean (Kocira et al., 2015a, 2017a, 2018b) and soybean (Kocira et al., 2018a). This is due to the fact that the increasing total content of phenolic acids leads to an increasing number of their functional groups, which are sequestrants of free radicals (Pantelidis et al., 2007; Du et al., 2009).

Apart from various active compounds of the tested biostimulants, as well as their doses and concentrations, soybean productivity and seed quality were also determined by environmental factors occurring in the growing period (Kocira et al., 2017b). It needs to be emphasized that unbeneficial conditions appearing in the period of plant growth activate multiple defense systems of plants. Under such circumstances, plants tend to save energy and water reserves and their vital functionals are sustained from their own reserves. The appearance of stress factors in the growing period induces physiological changes in plants, which close their stomata to prevent moisture loss and to retard the processes of photosynthesis, leading to the inhibition of metabolic processes (Spiekers and Pothast, 2004). The differences noted in the results from our study could be due to the changes in the average air temperature and precipitations in particular years of the field experiment that were stress factors to the plants. Similar observations were made by Grabowska et al. (2012) whose study demonstrated the effectiveness of biostimulants to depend not only on carrot cultivars but, most of all, on meteorological conditions in the period of plant growth and development. Such a great impact of these conditions results from the fact that biostimulants are systemic preparations, the active substances of which have to be transported to the active sites of plant tissues. Hence, their effectiveness is also determined by the hydrothermal conditions occurring after their application at the appropriate stages of plant growth (Kolomaznik et al., 2012).

The concept of determining the contents of cell wall fractions, i.e., NDF and ADF, has been proposed by Van Soest in the USA. He assumed that feedstuffs are composed of cell walls [cell wall constituents (CWC)] and the contents of cells [cell contents (CC)]. In the analytical system proposed by Van Soest, cell wall constituents, determined as NDF and ADF, were factors that reduced feed intake, digestibility, as well as energy value (Brzóska and Sliwinski, 2011). A surprising outcome of our study was a decrease in the content of the NDF fraction noted in all combinations in which plants were treated with biostimulants, compared to the control samples. A negative value of the difference between the NDF content in combinations with biostimulants and control samples (ABT-C) was determined for all analyzed seeds. However, the highest value of this index was observed after Atonik application. The statistical analysis of DNF contents demonstrated no significant differences between biostimulants in their effect on the total content of lignin, hemicellulose, and cellulose in soybean seeds. From the chemical point of view, the ADF is a sum of lignin and cellulose. Soybean plants responded to the treatment with higher concentrations of the biostimulants with an increased content of this fraction of dietary fiber (ADF). The statistical analysis of results demonstrated a positive value of the index of biostimulant effect on the ADF content (ABT-C) in the analyzed seeds. The highest value of this index was determined in soybean seeds after the foliar application of the Tytanit preparation.

Lignins belong to nondigestible phenolic compounds that are accumulated in cell walls of a plant with aging and are responsible for reducing the digestibility of its cell wall carbohydrates. Nevertheless, they are important for plant durability. Our study showed that the foliar application of biostimulants with different compositions led to an increased ADL content in soybean seeds. As reported by Chen et al. (2006), such an increase may be due to the contents of individual phenolic acids, particularly of ferulic acid that is a precursor of structural polymers of biosynthesis, such as lignins. The greatest increase in the lignin content was demonstrated in seeds of soybean treated with Tytanit. It needs to be emphasized that lignins are constituents of cell walls, and when combined with cellulose, impart mechanical resistance to plants. However, from the viewpoint of the defense mechanisms of a plant, their effect is mostly associated with the enhanced capability for counteracting the effects of adverse biotic factors (Bennett and Wallsgrove, 1994). Their high content in plants is a factor that determines their constitutive resistance (Poorter et al., 2004). In addition, their presence enhances the inducible resistance of plants, associated with the formation of the so-called lignin barriers that impair the expansion of pathogenic microorganisms (Rengel et al., 1994; Karolewski and Jagodzinski, 2013).

The hemicellulose contents determined in our study were observed to decrease upon the foliar application of both biostimulants. The calculated value of the ABT-C index was negative in all analyzed combinations. The greatest decrease in the HCEL content, compared to the control sample, was observed after the application of Atonik.

The difference between the contents of the acid fraction (ADF) and the lignin fraction (ADL) of dietary fiber provides information about the cellulose (CEL) content. A high content of hemicelluloses is a very beneficial phenomenon, considering their positive effect on physiological processes resulting from their capability to swell and absorb water in the lumen of the gastrointestinal tract of humans and monogastric animals. In addition, they offer optimal conditions for bacterial proliferation in colonic lumen (Piesiewicz and Bartnikowska, 1997).

Our study demonstrated that the analyzed biostimulants caused significant differences in the CEL content in soybean seeds. After their application, the ABT-C index attained negative values.

According to Silva et al. (2016), biostimulants may affect the content and technological characteristics of dietary fiber. However, as emphasized by Wang et al. (2010), fiber biosynthesis is an extremely complex process determined by the nutritional status of plants and also by abiotic factors. The stage of biosynthesis is characterized by an increased production of gibberellin that has a direct impact on fiber micronaire, length, and strength (Wang et al., 2010). As reported by Silva et al. (2016), the foliar application of biostimulants may contribute to an increasing content of gibberellins in treated plants, thereby modifying the formation process of fiber and its fractions.

The results obtained for the soybean treated with biostimulants were subjected to a correlation analysis. Some statistically significant correlations (*p* = 0.05) were found between the selected features. A strong negative correlation was found between total protein content and plant height (*r* = −0.91), number of seeds (*r* = 0.72), and seed yield (*r* = −0.69) after the application of Tytanit. It may be suggested that plants converted their metabolism and used energy for the growth of stems, leaves, and pods (Baglieri et al., 2014) as well as for the accumulation of fat, which is the main storage material in soybean seeds, rather than for production of storage proteins. This statement may be also supported by a positive correlation found between plant height and yield as well as number of pods and number of seeds. Previously, similar observations were made by Kocira et al. (2017a) after the application of Atonik in bean cultivation. On the other hand, a strong positive correlation was found between total fat content and number of pods (*r* = 0.60), seed number (*r* = 0.68), and seed yield (*r* = 0.64). In the case of Atonik, the observed correlations were not so clear; however, similarly, a strong negative correlation was found between total protein content and plant height (*r* = −0.86). An increased growth of plant was also confirmed by a significant correlation between yield and NDF (*r* = 0.66). Seed number was strongly positively correlated with yield. Most importantly, in plants treated with this biostimulant (regardless of combination), the protein and lipid contents were positively correlated with the 1000-seed weight. Such correlations were not found for Tytanit. The reducing power of both control samples and samples treated with biostimulants was positively correlated with the phenolics content. It is in agreement with the results of previous studies by Peng et al. (2017) and Lazo-Vélez et al. (2018) with regard to the antioxidative capacity of soybean. To sum up, biostimulants increase plant growth and positively influence plant productivity, but in many cases their effects are negatively correlated with the content of storage materials. On the other hand, treatments with biostimulants increase the content of “pathogen-related” components such as phenolics or dietary fiber that are usually increased during the systemic response of plants to stress conditions (Zhao et al., 2005; Fujita et al., 2006).

Although biostimulants offer new possibilities and their use in agriculture is regarded as safe and beneficial for crop productivity, the exact mechanism of their action remains unknown (Chojnacka, 2015; Polo and Mata, 2018), especially whether the effect of their application on plants is not a consequence of their direct capability to regulate plant metabolism, and whether their action may be multioriented. The most important is, however, the fact that these are the biostimulants and not the hormones that improve the metabolic processes of plants without altering their natural pathways (Posmyk and Szafranska, 2016; Polo and Mata, 2018). Due to the limited knowledge about the mechanisms of action of individual preparations, the inference from research still remains in the sphere of speculations and hypotheses. It is generally believed that biostimulants induce the growth and development of plants since seed germination through the entire ontogenesis. Their positive impact on plant metabolism is manifested in the enhanced synthesis and activity of plant hormones and in the activation of growth and development of the root system, contributing to better uptake, translocation, and retention of macro- and microelements and additionally determining crop productivity and yield quality (Basak, 2008; Calvo et al., 2014).

The results of our experiment demonstrated a positive effect of the biostimulant Tytanit on the physiological processes in plants that contributed to the improvement in their growth, development, and yield, and this effect was attributable to the stimulation of the activity of some enzymes (catalase, peroxidase, lipoxygenase, and nitrate reductase), enhanced activity of ferric ions in cells, synthesis of assimilation pigments, and higher rate of nutrient uptake in the case of the biostimulant based on titanium (Carvajal and Alcaraz, 1998; Grenda, 2003).

The exact mechanisms activated by this biostimulant are, however, difficult to identify also due to the fact that titanium is one of the so-called components beneficial to plants, i.e., chemical elements that improve the health status of a plant organism, although this organism may grow and develop well without them (Bartnik et al., 2017; Lyu et al., 2017). A few hypothetical theories concerning the mechanism of Ti action in plants have been proposed in literature. These theories have suggested that the biological effects of Ti are based on inducing the defense mechanisms of a plant organism against Ti. In addition, a low dose of this element has been demonstrated to enhance the defense mechanisms, whereas a high one (toxic) to suppress them (the hormesis effect) (Carvajal and Alcaraz, 1998; Hrubý et al., 2002; Bartnik et al., 2017).

Carvajal and Alcaraz (1998) have postulated a hypothesis of Ti action through Fe activity based on their own experimental data and findings of other authors. Clarkson and Hanson (1980) have demonstrated an increase in the Fe^2+^ content in leaves, fruits, chloroplasts, and chromoplasts after the foliar application of Ti (IV) ascorbate. Considering the above, Carvajal and Alcaraz (1998) have concluded that Fe^2+^ is the metabolically active form of iron and a mobile fraction in plants (Uren, 1984). Earlier investigations conducted by Mehrotra et al. (1976) and Patel et al. (1977) proved that the total Fe content in chlorotic plants is higher than in green plants, most likely due to the reduction of the Fe^2+^ content. Based on this, Carvajal and Alcaraz (1998) have advanced a hypothesis that the activity of Ti-induced enzymes increases directly (peroxidase and catalase) or indirectly (reduction of nitrates) in response to the presence of Fe. In addition, the fact that Ti supports a high number of vital processes of plants has prompted these authors to conclude that it would be slightly likely that this effect is due only to the presence and action of Ti in the established metabolic pathway.

The first suggestions related to the Ti activity mediated by Fe have been presented in the 1980s by Simon et al. (1988) who, after determining a higher chlorophyll content in Chlorella treated with Ti, signalized the possible positive effect of titanium on increased retention of magnesium and iron through the stimulation of the biosynthesis of pigments and prevention of their enzymatic degradation. In turn, Kiss et al. (1985) and Leidi et al. (1991) reported that iron affected the photochemical capability of plants that is associated with the electrolyte transport chain and chlorophyll synthesis. Based on these conclusions, Carvajal and Alcaraz (1995) conducted analyses of Fe^2+^ that confirmed that the increase in the content of the active Fe^2+^ fraction might be induced by a low redox potential of Ti^3+^/Ti^4+^. In such a case, most of the advanced hypotheses and experimental results obtained may be consistent with this explanation. However, according to Carvajal and Alcaraz (1998) and Lyu et al. (2017), the induction of the activity of other metals or other than the speculated mechanism of action is also likely. Dumon and Ernst (1988) suggested an alternative explanation of the mechanisms and effects induced by Ti. In their opinion, these effects may be due to the increased availability of elements as a result of increased direct or indirect possibility of processes of their absorption (different forms of ATPases). However, according to Carvajal and Alcaraz (1998) and Lyu et al. (2017), this assumption cannot explain why the foliar application of preparations with titanium ensures better effects than their application to the rhizosphere.

The latest theory proposed by Lyu et al. (2017) assumes that the beneficial role titanium plays in plants consists mainly in the interactions with other nutritive elements, Fe in particular. This hypothesis is not novel but was extended by these authors with a conclusion that titanium and iron may both form synergistic or antagonistic compounds. When plants are deficient in iron, titanium may induce the expression of genes associated with iron uptake, i.e., with increasing its capture and retention that consequently leads to plant growth improvement, because plants may have proteins that are capable of specific or nonspecific binding with titanium. When the content of Ti is high in plant tissues, titanium may compete with iron for ligands or proteins. The phenomenon of competition may be hazardous to plants due to Ti phytotoxicity at its high levels in plants (Ghooshchi, 2017; Lyu et al., 2017).

It should be emphasized that the diversity of conducted investigations as well as presented speculations and hypotheses indicates the incomplete understanding of the mechanism of Ti action and shows that all presented theories have both strengths and weaknesses. For this reason, further research is required to determine the mechanism of Ti actions.

Atonik has been used for many years in the cultivation of various crops worldwide; however—as in the case of Tytanit—the knowledge on the mechanisms of its action is still sparse. The first literature reports have indicated that its positive effect on crops results from the enhanced flow of the cytoplasm, leading to an increased rate of molecule transport both within and between tissues (Yamaki et al., 1953; Kudrev, 1969; Wilson and Kaczmarek, 1993).

Atonik application has also been demonstrated to affect the inhibition of IAA oxidase, which activates the enhanced natural synthesis of endogenous auxins in plants (Stutte and Clark, 1990; Djanaguiraman et al., 2004b, 2005). This inhibition results most of all from the fact that the phosphorylated form of p-nitrophenolate, being a substrate for phosphates, increases IAA activity (Davies, 1987), by acting similarly to ATP (Kurzumi et al., 1990). In addition, the use of Atonik may influence the nitrogen metabolism in plants that is manifested by the enhanced activity of nitrate reductase (Sharma et al., 1984; Gawronska et al., 2008; Przybysz et al., 2014). In addition, Atonik evokes a positive effect on the production of proline and polyols—two important compatible metabolites engaged in antistress mechanisms (Djanaguiraman et al., 2004a, 2009).

According to Djanaguiraman et al. (2010) and Przybysz et al. (2014), the active compounds of Atonik affect most of all the metabolism of reactive oxygen species (ROS). The disturbance of the balance between ROS generation and metabolism in plants leads to the induction of oxidative stress as a result of increased contents of H_2_O_2_, OH^−^, and O^2−^. A study conducted by Djanaguiraman et al. (2010) proved that the level of ROS decreased significantly in Atonik-treated plants and might indicate that this biostimulant activates also the defense mechanisms of plants, owing to which they may cope with the oxidative stress by enhancing the activity of enzymes of their antioxidative system and by increasing their total antioxidative capability (Djanaguiraman et al., 2004a, 2005, 2009; Wrochna et al., 2008). The above changes occur as a result of the increased activity of enzymatic [catalase (CAT), superoxide dismutase (SOD)] and nonenzymatic antioxidants [ascorbate (AA), glutathione (GSH), ascorbate peroxidase (APX), Navabpour et al., 2003; Zimmermann and Zentgraf, 2005]. It was also found that the increased AA level in Atonik-treated plants may result from the role of ascorbate in the reduction of hydrogen peroxide, in quenching a singlet oxygen or the regeneration of reduced alpha-tocopherol (Bartoli et al., 1999; Smirnoff and Wheeler, 2000). As reported by Mayak et al. (1981), superoxide anions, formed in higher numbers under stress conditions to plants, induce degradation of phospholipids, whereas fatty acids released during this degradation undergo peroxidation (Simon, 1974). However, according to Djanaguiraman et al. (2010), low contents of O_2_ and H_2_O_2_ in plants treated with nitrophenols may result from the entrapment of free radicals by phenolic compounds, because phenols are capable of inhibiting the oxidation of lipids and proteins by donating phenolic hydrogen to a free radical (Aruoma et al., 1993; Halliwell et al., 1995). Stereophonic effects of phenols and phenoxyl radicals of Atonik are mainly due to their reactivity with radicals (Burton et al., 1985). Mechanisms of action in which a hydrogen atom of phenols is transferred to a radical may proceed in two different pathways. The first one involves the transfer of a hydrogen atom, whereas the other, the transfer of an electron with the use of protons (Mayer et al., 2002). As reported by Frankel et al. (1996) and Jang et al. (2007), the effect of Atonik on ROS is mainly linked with the antioxidative properties of the supplied phenols that may act as scavengers of radicals or may split radical chains, thereby extinguishing the strongly oxidizing free radicals (Stadler et al., 1995; Moran et al., 1997). These speculations on the mechanism of Atonik action were confirmed by the results of a research conducted by Djanaguiraman et al. (2010), who demonstrated that crops sprayed with nitrophenols had a higher capability for ROS elimination through a higher activity of peroxidase (POX) induced by a greater availability of the substrate in the form of guaiacol (active substance of Atonik preparation). In addition, Zancani and Nagy (2000) proposed a hypothesis that POX is effective as a system for H_2_O_2_ capture in plant vacuoles in the presence of phenolic compounds.

The latest research findings indicate, however, that plants' response to the treatment with Atonik is probably underlaid by the modification of the expression profile of genes linked with the defense mechanism (Przybysz et al., 2014). So far, however, only few works have confirmed this hypothesis. Cambri et al. (2008) presented the results of a study on the effect of biostimulants on gene expression. They demonstrated the induction of the expression of some genes involved in the defense mechanisms of *Arabidopsis thaliana* L. plants cultivated under conditions of salt stress. In turn, an experiment carried out by Gawronska et al. (2008), with the use of the micromatrix technology, proved a change in the gene expression profile in plants treated with Atonik. Based on the analysis conducted with bioinformatic tools, these authors have demonstrated that the biostimulant based on nitrophenolic compounds modified the expression of 801 genes in *Arabidopsis thaliana* L. plants (748 genes were upregulated, while only 53 were downregulated). In addition, the experiment of Gawronska et al. (2008) has demonstrated that the upregulated genes included mainly those responsible for protein metabolism, transcription, transport, electron transport or energy pathways, developmental processes, and response to stress as well as abiotic and biotic stimuli. Presumably, further studies in this field will allow for the acquisition of more in-depth knowledge on the mechanisms of action of this biostimulant.

Although biostimulants have been used in cultivation for many years because, in many cases, they may improve crop resistance to environmental disturbances, the priority goal of present-day investigations on these preparations should be to better understand the cause-and-effect mechanism of their action (Van Oosten et al., 2017). According to Van Oosten et al. (2017) and Povero et al. (2016), only proper and complete understanding of these mechanisms will allow us to design the next generation of biostimulants with the desired functional properties. The understanding of the specific mechanisms on the basis of hypotheses will not be possible without merging knowledge and tools from many scientific disciplines including agronomy, biology, chemistry, or genetics.

## Conclusions

The type of biostimulant, number of its applications, and its concentration significantly modified the biometric traits, crop productivity, and yield quality as well as the nutraceutical and antioxidative potential of soybean seeds. It was demonstrated that while positively affecting the growth of plants and seed yield, the Atonik and Tytanit preparations caused only a little decrease in the protein and fat contents in soybean seeds. In addition, a positive effect was noted of the double application of these biostimulants in their higher concentration on soybean seed number and seed yield. The tested biostimulants increased the antioxidative activity of soybean seeds expressed by the total content of phenols, flavonoids, and anthocyanins, and by the reducing power. The greatest increase was observed after the application of Tytanit. Dietary fiber fraction analysis demonstrated that the use of biostimulants caused an increase in the acid-detergent fiber and lignin contents in soybean seeds, with the greatest increase observed again upon the use of Tytanit. Simultaneously, a decrease was noted in the neutral-detergent fiber, cellulose, and hemicellulose contents in soybean seeds from all combinations treated with the tested preparations. Results obtained in our study point to the need of continuing and extending research with the aim to identify responses of various crops on the treatment with biostimulants based on different active substances. Such investigations would allow enriching knowledge on the mechanisms of action of such preparations.

## Author contributions

AS and SK conceived and supervised the whole study, analyzed the plant material, and wrote the manuscript. AK carried out the field experiment and wrote the manuscript. EC and MŚ analyzed the plant material and contributed to the drafting of the manuscript. RK performed the statistical analysis. EL gave experimental advice and contributed to the drafting of the manuscript. MK carried out the field experiment. TO analyzed the plant material. All the authors read and approved the final manuscript.

### Conflict of interest statement

The authors declare that the research was conducted in the absence of any commercial or financial relationships that could be construed as a potential conflict of interest.
